# Hydrogen Bonding Phase-Transfer Catalysis with Alkali Metal Fluorides and Beyond

**DOI:** 10.1021/jacs.2c00190

**Published:** 2022-03-16

**Authors:** Gabriele Pupo, Véronique Gouverneur

**Affiliations:** Chemistry Research Laboratory, University of Oxford, 12 Mansfield Road, Oxford OX1 3TA, U.K.

## Abstract

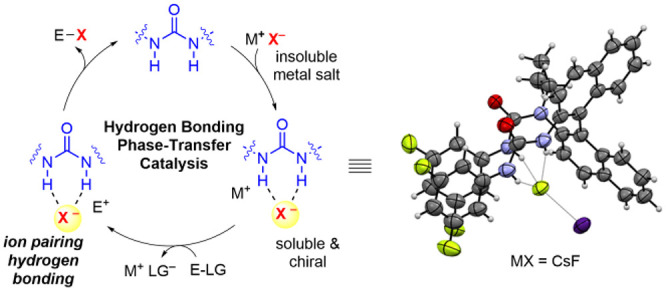

Phase-transfer catalysis (PTC) is one of the most powerful catalytic manifolds for asymmetric synthesis. Chiral cationic or anionic PTC strategies have enabled a variety of transformations, yet studies on the use of insoluble inorganic salts as nucleophiles for the synthesis of enantioenriched molecules have remained elusive. A long-standing challenge is the development of methods for asymmetric carbon–fluorine bond formation from readily available and cost-effective alkali metal fluorides. In this Perspective, we describe how H-bond donors can provide a solution through fluoride binding. We use examples, primarily from our own research, to discuss how hydrogen bonding interactions impact fluoride reactivity and the role of H-bond donors as phase-transfer catalysts to bring solid-phase alkali metal fluorides in solution. These studies led to hydrogen bonding phase-transfer catalysis (HB-PTC), a new concept in PTC, originally crafted for alkali metal fluorides but offering opportunities beyond enantioselective fluorination. Looking ahead, the unlimited options that one can consider to diversify the H-bond donor, the inorganic salt, and the electrophile, herald a new era in phase-transfer catalysis. Whether abundant inorganic salts of lattice energy significantly higher than those studied to date could be considered as nucleophiles, e.g., CaF_2_, remains an open question, with solutions that may be found through synergistic PTC catalysis or beyond PTC.

## Introduction

**Scheme 1:**
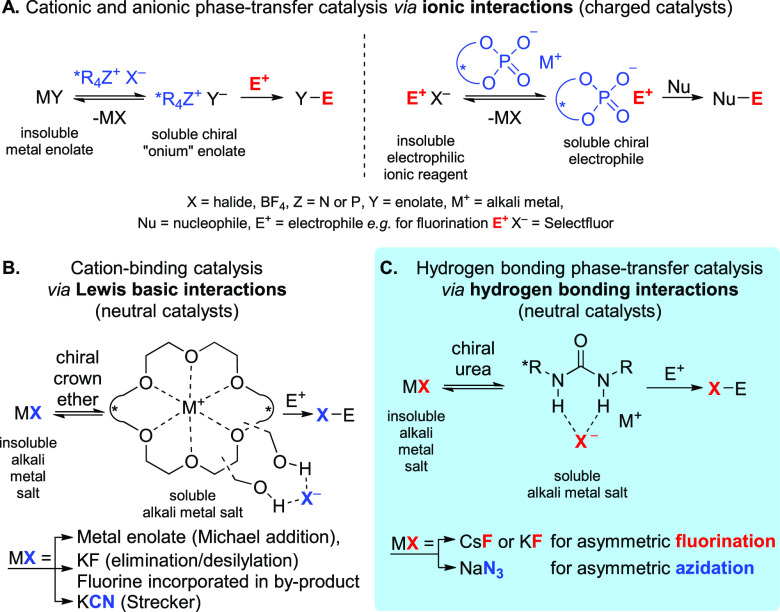
Asymmetric Phase-Transfer Catalysis via (A) Ionic Interactions (Charged Catalysts), (B) Lewis Basic Interactions (Neutral Catalysts) and Hydrogen Bonding Interactions, and (C) Hydrogen Bonding Interactions (Neutral Catalysts)

Phase-transfer catalysis (PTC) enables the rate enhancement of a reaction between molecules located in different phases.^1^ Since its discovery more than 50 years ago,^2^ PTC has evolved into a broadly applicable tool in both academia and industry and has been extensively applied to asymmetric synthesis.^3^ Traditional PTC employs lipophilic charged catalysts bearing chiral cations^3b^ or anions^4^ (Scheme 1A) and relies on ion pairing for interface crossing. An alternative strategy involves the use of neutral crown ethers to encapsulate the alkali metal cation of an inorganic salt, e.g., KF or KCN, thus generating a soluble nucleophilic anion whose reactivity can be tuned through hydrogen-bonding interactions (Scheme 1B).^5^ Despite these advances, the use of insoluble inorganic salts as nucleophiles in asymmetric catalysis largely remains an unsolved problem in solid–liquid PTC. Inorganic salts are often ideal in terms of safety, cost, and simplicity of handling, but their poor solubility in organic solvents has hampered applications in enantioselective transformations. This challenge became central to our research program.

In nature, enzymes harness inorganic salts through anion recognition^6^ (e.g., halides, nitrate, sulfate, or phosphate) based on electrostatic and/or hydrogen bonding interactions. With this knowledge, we hypothesized that the transport of inorganic salts from the solid phase into solution may be accomplished through hydrogen bonding interactions to anions. The nucleophilicity of the resulting hydrogen-bonded anion would be attenuated yet sufficient for ensuing transformations; moreover, asymmetric fluoride delivery may be within reach in the presence of a chiral H-bond donor catalyst. This line of thought led to the development of hydrogen bonding phase-transfer catalysis (HB-PTC), a new PTC manifold for the broader use of inorganic salts as reagents in asymmetric catalysis (Scheme 1C).^7^ Conceptually, under HB-PTC, a neutral chiral H-bond donor (e.g., urea) brings an insoluble and therefore unreactive alkali metal salt in solution, thus generating *in situ* a hydrogen-bonded chiral nucleophile with controllable reactivity. This species can then be intercepted with an appropriate electrophile in an asymmetric fashion through the formation of a chiral ion pair. Due to the importance in the pharmaceutical industry of compounds bearing fluorine on a stereogenic carbon,^8^ and our ongoing interest in the production of chiral fluorochemicals, our laboratory focused first on the activation of alkali metal fluorides for the asymmetric installation of C–F bonds on aliphatic compounds.

In this Perspective, we discuss the workflow that led to the development of HB-PTC with first a description of hydrogen-bonded fluoride complexes derived from alcohols and ureas and the impact of hydrogen bonding on fluoride reactivity in non-asymmetric transformations. We then discuss how, in our laboratory, these studies were foundational to the development of HB-PTC and its application to enantioselective fluorination reactions with alkali metal fluorides. The application of HB-PTC to inorganic salts other than metal alkali fluorides is also discussed, with an outlook on future challenges and opportunities.

## The Fluorinase Enzyme and Hydrogen-Bonded Fluoride Complexes

**Scheme 2:**
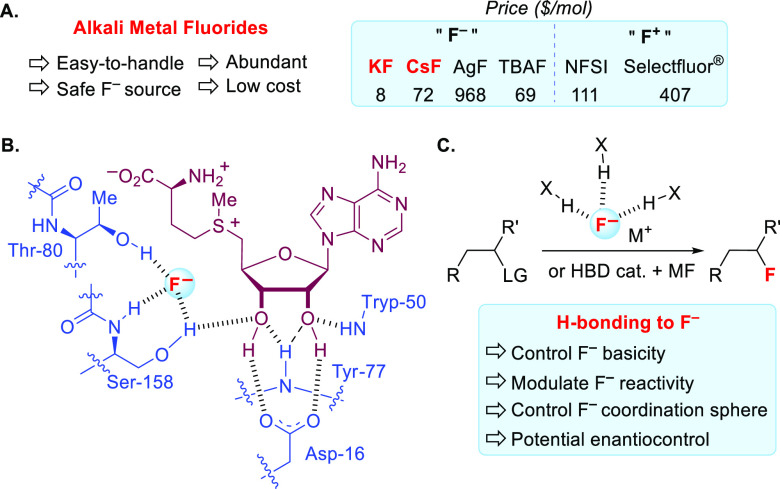
(A) Alkali Metal Fluorides as Fluorinating Reagents,^20^ (B) Schematic Representation of the Active Site of the Fluorinase Enzyme (*Streptomyces cattleya*), and (C) Nucleophilic Fluorinations Promoted/Catalyzed by H-Bond Donors LG = leaving group, MF = fluoride salt.

Despite its rarity in natural products, fluorine is fundamental to our daily lives, with as many as 35% of agrochemicals, 20–25% of marketed drugs, and numerous anesthetics and materials containing one or more fluorine atoms.^8a,9^ Fluorine substitution is a tactic extensively exploited in drug discovery to modulate lipophilicity, metabolic stability, and bioavailability^10^ and has also found numerous applications in ^19^F magnetic resonance imaging (MRI).^11^ Furthermore, the radioisotope ^18^F is central to positron emission tomography (PET), a powerful non-invasive molecular imaging technology that facilitates drug discovery, diagnosis, and personalized healthcare.^12^ New and more efficient methods to incorporate fluorine (^19^F or ^18^F) are therefore continuously in demand, particularly late-stage protocols with broad applicability.^13^ Electrophilic reagents such as NFSI or Selectfluor have been successfully employed in C(sp^2^) and C(sp^3^) fluorinations, including asymmetric variants.^13c,14^ Despite their extensive use, these reagents suffer from poor atom economy, limited reactivity, and high cost. In contrast, nucleophilic fluorine sources, and more specifically low-cost alkali metal salts (CsF and KF), are atom economical and easy to handle compared to alternative reagents such as toxic DAST or HF that require safety hazards management (Scheme 2A).^15^ For radiochemistry, [^18^F]fluoride is preferred over electrophilic ^18^F reagents derived from [^18^F]F_2_ because these “F^+^ ” sources are difficult to produce and suffer from low molar activity.^12^ Despite these advantages, the poor solubility of metal alkali fluorides (lattice energy: CsF, 744 kJ/mol; KF, 820 kJ/mol)^16^ and high Brønsted basicity in polar aprotic solvents have discouraged their use in asymmetric catalysis. Polar protic solvents capable of H-bonding interactions (e.g., alcohols or water) with fluoride have been considered to help solubilization at the expense of reduced nucleophilicity. Encapsulation of the metal by crown ethers also releases soluble “naked” fluoride, but the simultaneous enhancement of fluoride basicity leads to unwanted side reactions such as elimination or the cleavage of base-labile groups. These challenges led numerous groups to generate soluble F^–^ of controllable reactivity from alkali metal fluorides by harnessing the power of H-bonding interactions,^17^ an approach that our laboratory pursued being guided by the fluorinase enzyme.

### The Fluorinase Enzyme

Twenty years ago, O’Hagan and co-workers reported the discovery of a fluorinase enzyme and its mode of action for C–F bond formation (Scheme 2B).^18^ Mechanistically, the active site features a network of three hydrogen bonding interactions around fluoride that compensates for the penalty incurred by desolvation, a necessity considering the high hydration free energy of fluoride (∼440 kJ/mol).^19^ Importantly, this enzymatic heteroleptic tricoordinated fluoride complex is sufficiently nucleophilic to attack the positively charged sulfonium substrate (*S*-adenosyl-l-methionine), offering 5′-fluorodeoxyadenosine (5′-FDA) upon nucleophilic substitution (S_N_2). This insight suggested to us that precisely arranged hydrogen bonding interactions around fluoride such as those found in the fluorinase enzyme could be utilized in organic synthesis to control fluoride reactivity, including favoring nucleophilicity over basicity. Moreover, through careful organization of the coordination sphere of fluoride, chiral H-bond donors could create an asymmetric environment for catalytic enantioselective fluorination (Scheme 2C).

### Alcohols as Modulators of Fluoride Reactivity

**Scheme 3:**
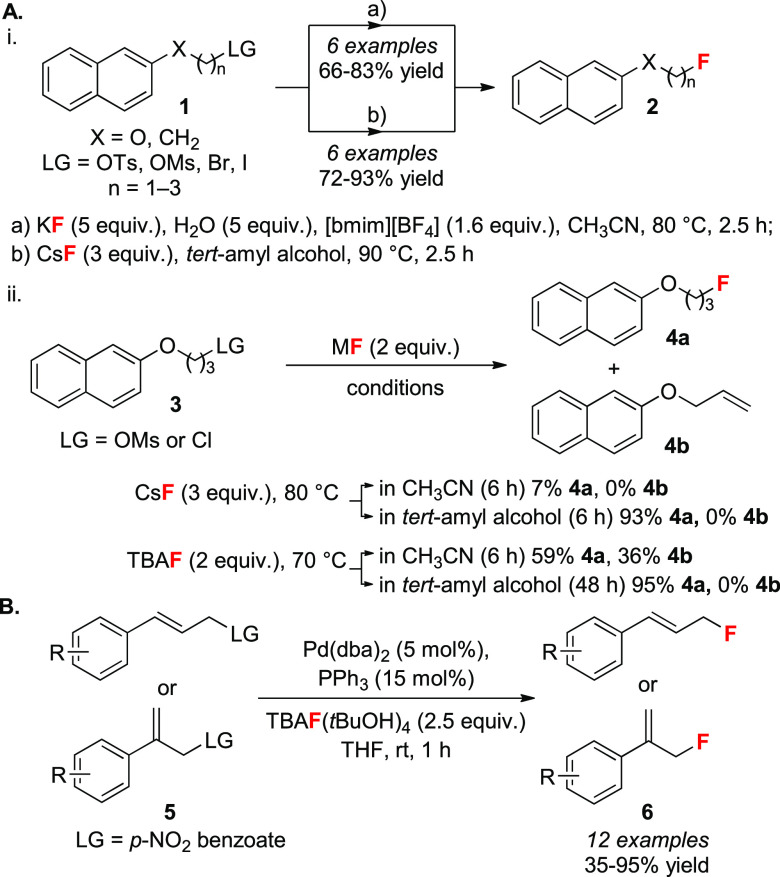
Early Examples of the Use of Tertiary Alcohols as Modulators of Fluoride Reactivity in Solution: (A) (i) S_N_2 Fluorinations Promoted by Ionic Liquid and H_2_O and (ii) S_N_2 Fluorinations in Tertiary Alcohols as Solvents, and (B) Use of TBAF(*t*BuOH)_4_ in Tsuji–Trost Allylic Fluorination LG = leaving group.

The first report studying the effect of H-bonding on fluoride reactivity was disclosed in 1994 by Yonezawa and co-workers, and focused on tetrabutylammonium fluoride (TBAF) complexes with alcohols.^21^ A model S_N_2 reaction served to demonstrate that the rate of fluorination of alkyl bromides increased with the size of the H-bond donor (*t*BuOH ≫ *i*PrOH > H_2_O). Almost a decade later, KF was successfully employed as a fluorinating reagent in the conversion of alkyl mesylates **1** to alkyl fluorides **2** by employing stoichiometric amounts of ionic liquid ([bmim][BF_4_]) in acetonitrile at elevated temperatures (100 °C) (Scheme 3A-i, conditions a).^22^ This concept was expanded to CsF with polymer-supported ionic liquids^23^ or ionic liquids bearing pending tertiary alcohols.^24^ The addition of 5 equiv of water as H-bond donor ensured higher yields as well as superior selectivity in favor of the fluorinated product for elimination-prone substrates. Kim and co-workers subsequently disclosed a protocol for the S_N_2 substitution of alkyl mesylates **1** using CsF (or [^18^F]TBAF) in tertiary alcohols (e.g., *tert*-butyl or *tert*-amyl alcohol) as solvents (Scheme 3A-i, conditions b).^25^ While H-bonding interactions lowered fluoride’s nucleophilicity, the solubility of CsF was improved,^25a^ and the selectivity for S_N_2 vs E2 (**4a** vs **4b**) increased,^25c^ even when TBAF was used instead of CsF (Scheme 3A-ii).^26^ The same group also reported the single-crystal X-ray structure of the TBAF(*t*BuOH)_4_ complex and its use for fluorination.^27^ The same reagent was successfully employed by our group in Tsuji–Trost-type allylic fluorination of *p*-NO_2_-benzoates and proved to be superior to both CsF and TBAF due to adequate nucleophilicity combined with low basicity and hygroscopicity (Scheme 3B).^28a^ The methodology was later extended to iridium-catalyzed fluorinations of allylic carbonates.^28b^ Very recently, anhydrous TBAF(*t*BuOH)_4_ was successfully employed as a fluorinating reagent in the radical fluorodecarboxylation of benzoic acids in acetonitrile; its use suppressed competing C–O reductive elimination observed when using TBAF.^29^

**Scheme 4:**
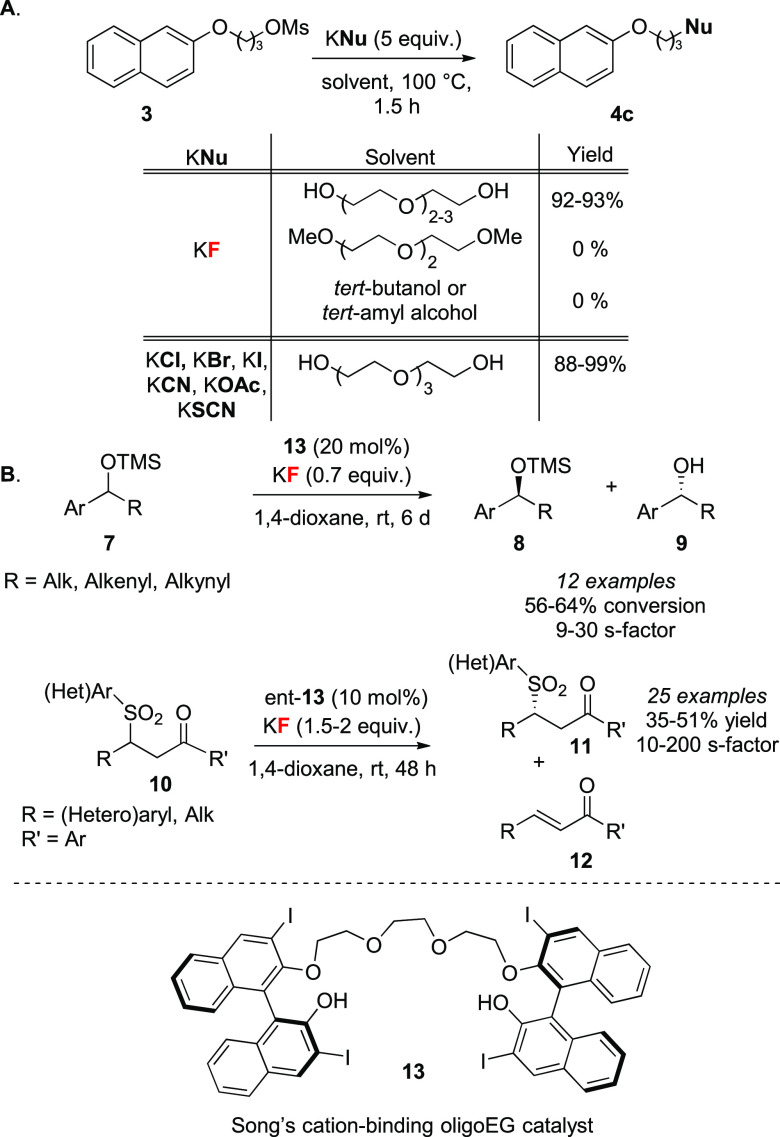
(A) Tri- and Tetraethylene Glycol as Solvent in Model S_N_2 Reactions and (B) Desilylative Kinetic Resolutions of Silylated Secondary Alcohols and Asymmetric Elimination with KF Alk = alkyl.

An early synergistic approach to fluorination with KF was reported by Lee, Chi, Song, and co-workers.^30^ Achiral polyethers with a pending alcohol (e.g., tri- or tetraethylene glycol) were employed as solvents in order to encapsulate the cation of an inorganic fluoride salt while simultaneously modulating the reactivity of fluoride and activating the electrophile through hydrogen bonding. The approach was validated with the fluorination of alkyl mesylate **3** with KF, which showed that these solvents enabled substitution, which did not occur in *tert*-butanol and *tert*-amyl alcohol (Scheme 4A). The importance of the terminal H-bond donors was underlined by a control experiment in which bis-methylated tetraethylene glycol did not lead to product formation. This approach was extended to other halogenations (Cl, Br, I), cyanation, acetylation, and thio cyanation reactions using the corresponding potassium salts.^30^ Soon after, chiral BINOL-derived polyether catalysts (e.g., **13**, Scheme 4B) proved to be highly selective in the desilylative kinetic resolution of silylated alcohols **7**.^31^ By employing KF as a base rather than a nucleophile, asymmetric β-eliminations of β-sulfonyl ketones **10** (Scheme 4B),^32^*anti*-*syn*-trihalides, and *anti*-*syn*-*anti*-tetrahalides were disclosed.^33^ This strategy was also applied to Strecker^5d^ and silylation reactions,^34^ yet no examples involving C–F bond formation reactions ensued.

**Scheme 5:**
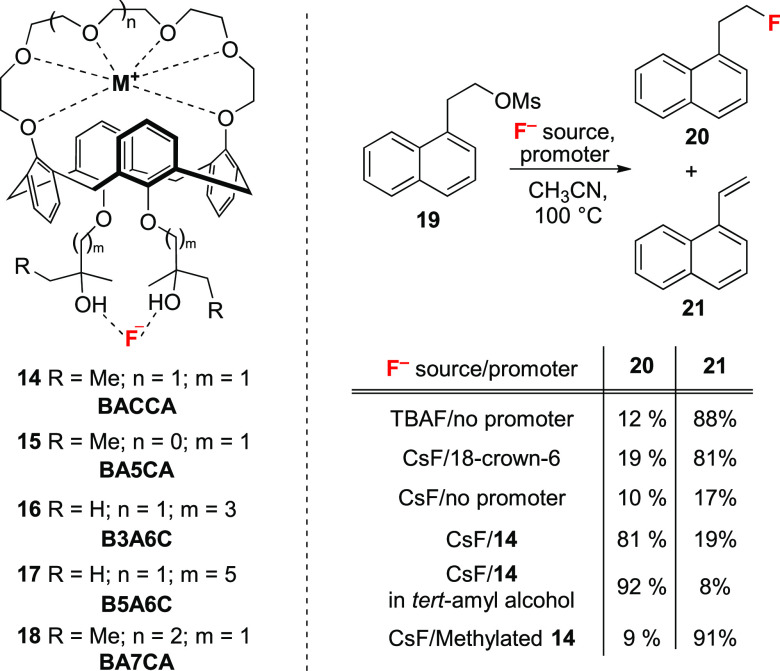
Fluorination of Alkyl Mesylates Using Calixarenes Functionalized with Crown Ethers and Tertiary Alcohols (1 equiv) as Promoters and TBAF or CsF (3 equiv) as Fluorinating Reagent

More recently, Pliego and co-workers disclosed an alternative synergistic approach in which an 18-crown-6 and a bulky diol (BDMB = 1,4-bis(2-hydroxy-2-propyl)benzene) were combined for the phase-transfer fluorination of alkyl bromides in acetonitrile with solid KF.^35^ In a complementary approach, Kim, Lee, and co-workers reported that crown-ether-strapped calix[4]arenes **14**–**18** can facilitate nucleophilic fluorination with CsF and KF (Scheme 5).^36^ BACCA (bis-*tert*-alcohol-functionalized crown-6-calix[4]arene, **14**) enabled the fluorination of alkyl mesylate **19**, a substrate prone to elimination, with S_N_2:E2 ratios higher than 10:1 when *tert*-amyl alcohol was employed as solvent (Scheme 5). When the alcohol groups were capped with a methyl group, the fluorinated product **20** was obtained in 9% yield, along with 91% yield of olefin **21**, thereby demonstrating the key role of the H-bond donor motif. Recently, the same authors further improved BACCA-type promoters by examining the size of the crown ether unit and the length of the alkyl chain bearing the tertiary alcohol.^37^ Superior reactivity was observed for the fluorination of alkyl mesylates with KF when BA5CA (*n* = 0, *m* = 1) (**15**), with its crown-5-calix[4]arene ideally suited for K^+^ binding, was employed instead of BACCA **14**. Furthermore, increasing the length of the alkyl spacer (B3A6C, **16**, and B5A6C, **17**) led to further charge separation and enhanced reactivity.

**Scheme 6:**
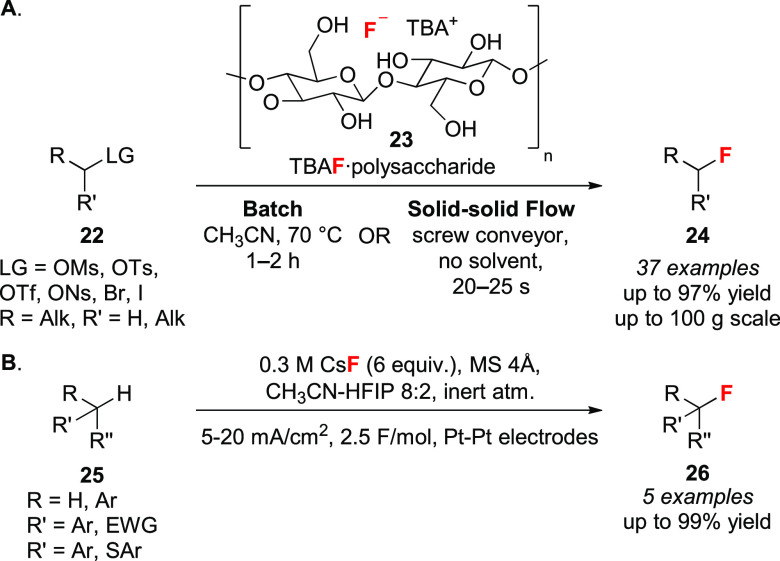
(A) Fluorination in Batch and Solid–Solid Flow Using Cellulose-Bound TBAF Complexes and (B) Electrochemical Fluorination with CsF and HFIP mA = milliamperes; F/mol = faraday per mole.

In 2020, the Dastager group reported the cellulose-supported TBAF complex **23** (Scheme 6A).^38^ This polymer-bound fluoride was superior to TBAF in terms of selectivity in selected S_N_2 reactions with alkyl halides **22** (S_N_2/E2 _TBAF_ = 0.02–0.5 vs S_N_2/E2 _polymer **23**_ = 1.41–6.1). The fluorination was scaled up to 100 g after which the cellulose promoter was recycled and reused upon filtration, drying, and further loading with TBAF(H_2_O)_3_. The reaction time was drastically reduced to 20–25 s applying solid–solid flow chemistry with a screw conveyor, a rare example of solid-state nucleophilic fluorination. An additional study by Inagi and co-workers demonstrated that the combined use of fluorinated alcohols and CsF enables the fluorination of activated C–H bonds (e.g., benzylic) under electrochemical conditions (Scheme 6B).^39^ This work reports the characterization of CsF/HFIP (HFIP = 1,1,1,3,3,3-hexafluoroisopropanol) and CsF/TFE (TFE = 2,2,2-trifluoroethanol) complexes by single-crystal X-ray diffraction (XRD) analysis.

**Scheme 7:**
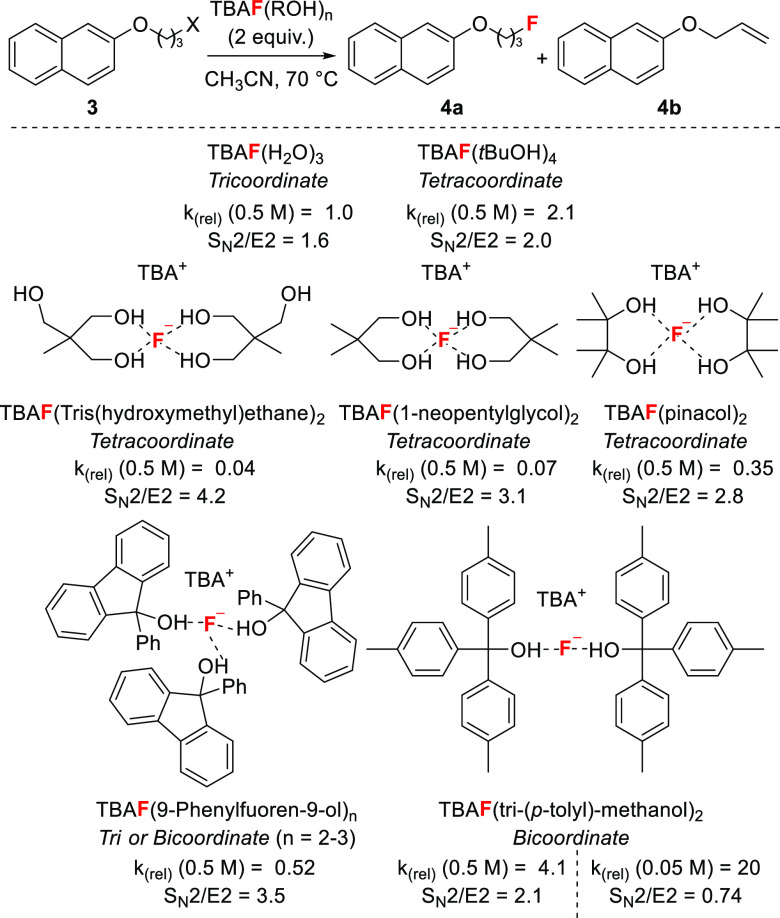
Solid-State Coordination Preference and Reactivity of Selected Alcohol–Fluoride Complexes *k*_(rel)_ = reaction rate relative to TBAF·(H_2_O)_3_.

Despite these important advances, the studies discussed so far provide limited insight into how the number and strength of H-bond contacts of alcohols to fluoride influence fluoride’s reactivity as a nucleophile or as a base. For this purpose, our group reacted a range of 1,2- and 1,3-diols as well as tri- and tetra-ols of increasing steric bulk with TBAF to prepare complexes of general structure TBAF(ROH)_*n*_ (*n* = 2–4) in order to study their reactivity (Scheme 7).^40^ These compounds, all characterized by XRD analysis, are easy-to-handle solids, with some much less hygroscopic than TBAF(*t*BuOH)_4_.^27^ Increasing the steric bulk and branching of the alcohol led to low coordination number and shorter H-bonds (e.g., donor–acceptor distance O---F for TBAF(pinacol)_4_ and TBAF(tritolylmethanol)_2_: 2.615–2.641 Å vs 2.499–2.554 Å). These complexes were tested as fluorinating reagents in a model S_N_2 fluorination reaction of alkyl mesylates or bromides **3**, and relative rates were measured. In this series, fluoride reactivity decreased and S_N_2 vs E2 selectivity improved when the number of H-bond contacts to fluoride increased from two to four (Scheme 7).

Keeping the number of H-bond contacts constant, our next objective was to quantify how fluoride reactivity can be fine-tuned through precise control of the strength of hydrogen bonding interactions. This study was achieved with a range of differently substituted 1,3-diarylureas.

### Ureas as Modulators of Fluoride Reactivity

**Scheme 8:**
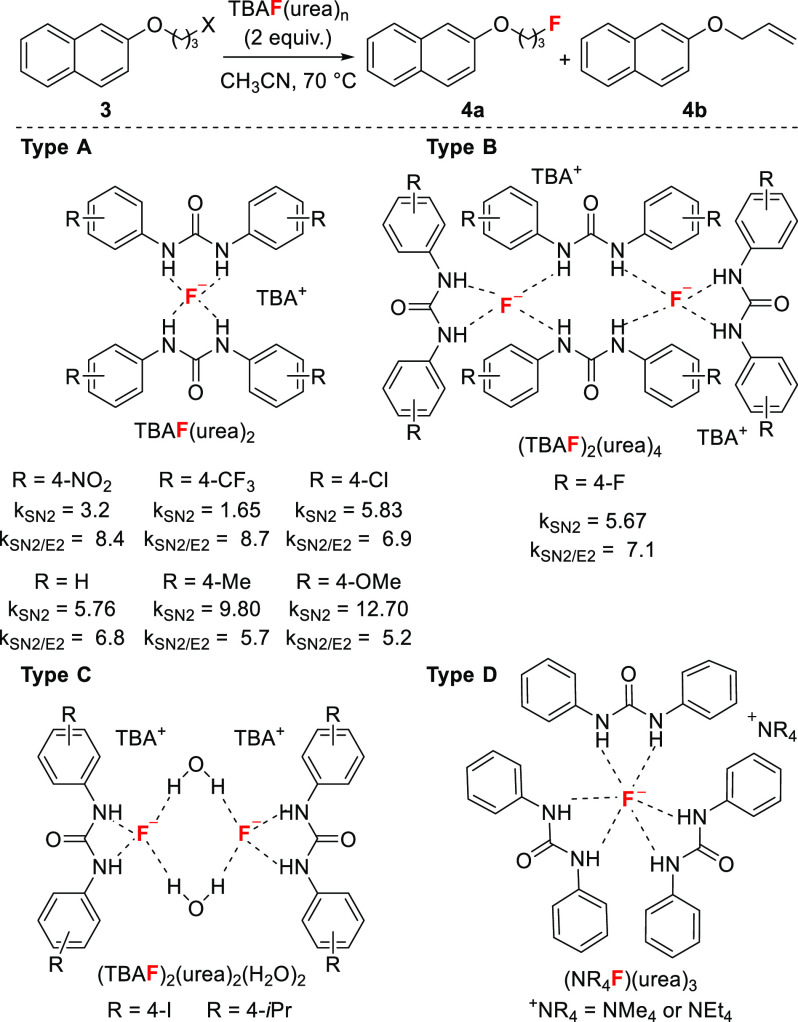
Structure and Reactivity of 1,3-Diarylurea–Fluoride Complexes *k*_SN2_ = reaction rate of S_N_2 fluorination (×10^–5^ M^–1^ s^–1^); *k*_SN2/E2_ = ratio of the rates of S_N_2 fluorination and E2 elimination. The values for TBAF(H_2_O)_3_ are *k*_SN2_ = 375 and *k*_SN2/E2_ = 1.6.

In 2016, we undertook a detailed study aimed at correlating the structure of urea–fluoride (UF) complexes with their reactivity for nucleophilic fluorination.^41^ We chose 1,3-diarylureas that are commonly employed as anion receptors and organocatalysts, because their electronics can be easily modified through aryl substitution.^6,42^ Eighteen 1,3-diarylurea–fluoride complexes were synthesized in high yields (77–99%) and recrystallized to afford single crystals suitable for XRD analysis (Scheme 8). For three complexes (R = 4-*n*-Pr, 4-Cl, 4-F), large crystals enabled neutron diffraction analysis, thus allowing the accurate measurement of NH···F distances (1.634–1.825 Å). A rich diversity of coordination modes was observed consisting of four types: (i) TBAF(urea)_2_, in which two ureas bind a single fluoride anion (Type A); (ii) (TBAF)_2_(urea)_4_, in which four ureas bind two fluoride anions (Type B); (TBAF)_2_urea_2_(H_2_O)_2_, in which two ureas are bound to two distinct fluoride anions, which are bridged by two molecules of water (Type C); and (iv) NR_4_F(urea)_3_, with R = Me, Et, in which three ureas coordinate the fluoride anion (Type D). Titration experiments in acetonitrile (^1^H NMR and UV–Vis spectroscopy) supported the presence of H-bond interactions with F^–^ in solution with the strength of H-bonding for these complexes being tunable through aryl substitution of the 1,3-diarylureas. Kinetics studies carried out on a model reaction of alkyl halides, demonstrate that tetracoordinated complexes of Type A are significantly less reactive than alcohol–fluoride complexes^41^ but are more selective for S_N_2 vs E2 (Scheme 8). Within this series, fluoride complexes derived from ureas bearing electron-withdrawing groups are less nucleophilic but display superior S_N_2 vs E2 selectivity than H-bonded complexes derived from ureas featuring electron-donating groups on the aryl moieties. These observations corroborate solid-state and titration experiments which indicate that depleted electron density on the aryl ring of the urea leads to shorter H-bond contacts with F^–^ and increased H-bonding strength, resulting in attenuated fluoride reactivity.

With the knowledge that the coordination sphere of H-bonded fluoride complexes can be precisely organized to control reactivity and S_N_2 vs E2 selectivity, the development of a catalytic asymmetric fluorination became a priority. This represented a significant challenge, not least because urea-bound fluoride complexes are significantly less reactive than their parent fluoride source, TBAF(H_2_O)_3_. To escape this impasse, we considered using alkali metal fluorides because these salts are insoluble and unreactive in organic solvents, thereby suppressing background reactivity. To be successful, this strategy would require that the chiral urea acts as phase-transfer agent to bring solid metal alkali fluoride into solution in the form of a soluble and reactive chiral urea-bound fluoride complex. In this scenario, the H-bonds network around fluoride would create the necessary chiral environment for asymmetric fluorination as well as enable control over S_N_2 vs E2 selectivity. With this reasoning, HB-PTC was born.

## Fluorination via Solid–Liquid Phase-Transfer: Hydrogen Bonding Phase-Transfer Catalysis with Alkali Metal Fluorides

### Asymmetric Nucleophilic Fluorination with CsF—Desymmetrization of Episulfonium Salts

**Scheme 9:**
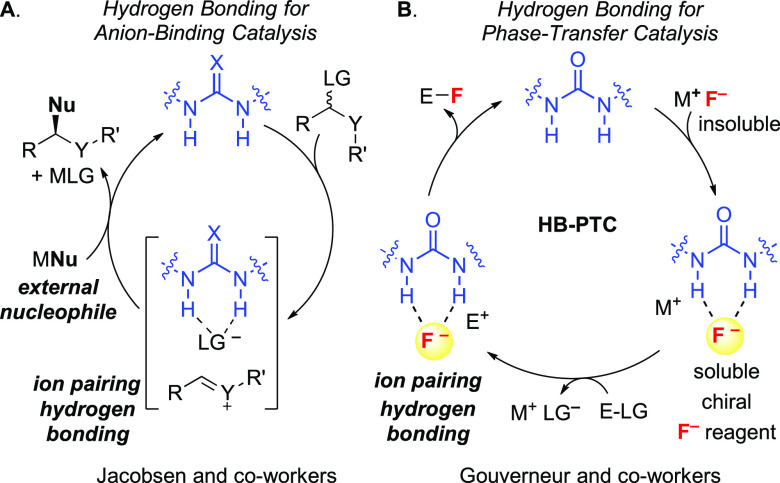
Hydrogen Bonding Interactions for (A) Anion-Binding Catalysis and (B) Phase-Transfer Catalysis E^+^ = electrophile; Nu = nucleophile; LG = leaving group.

Anion abstraction from organic molecules has been extensively explored in homogeneous catalysis using H-bond donors.^43^ Typically, an electrophile suitably armed with a leaving group, e.g., halide, is activated with a chiral H-bond donor catalyst via halide abstraction, leading to a chiral ion pair that reacts with an external nucleophile (Scheme 9A). In this scenario, the catalyst-bound halide serves as a chiral counteranion for asymmetric induction. For example, H-bonded fluoride complexes derived from chiral H-bond donor catalyst were featured in enantioselective acylations of silyl ketene acetals with benzoyl fluorides,^44^ and asymmetric desilylations or eliminations (Scheme 4B).^31−33^ Reactions in which the catalyst-bound anion is itself the nucleophile are scarce, in part due to attenuated nucleophilicity.^45^ Notable studies have been reported by Jacobsen and co-workers who reported the thiourea-catalyzed asymmetric ring-opening of aziridines with hydrogen chloride,^46^ and more recently the desymmetrization of oxetanes with TMSBr and a chiral squaramide as catalyst.^47,48^

Departing from homogeneous catalysis, we considered a PTC strategy to enable the use of metal alkali fluoride in enantioselective fluorination (Scheme 9B).^7a^ In this scenario that we coined hydrogen bonding phase-transfer catalysis (HB-PTC), a chiral H-bond donor acts as phase-transfer agent to bring solid, e.g., CsF, in solution as a chiral hydrogen-bonded fluoride complex capable of ion pairing ({urea·F^–^}{E^+^}) with a cationic electrophile E^+^. Upon fluoride delivery, formation of the enantioenriched alkyl fluoride closes the catalytic cycle with regeneration of the catalyst.

**Scheme 10:**
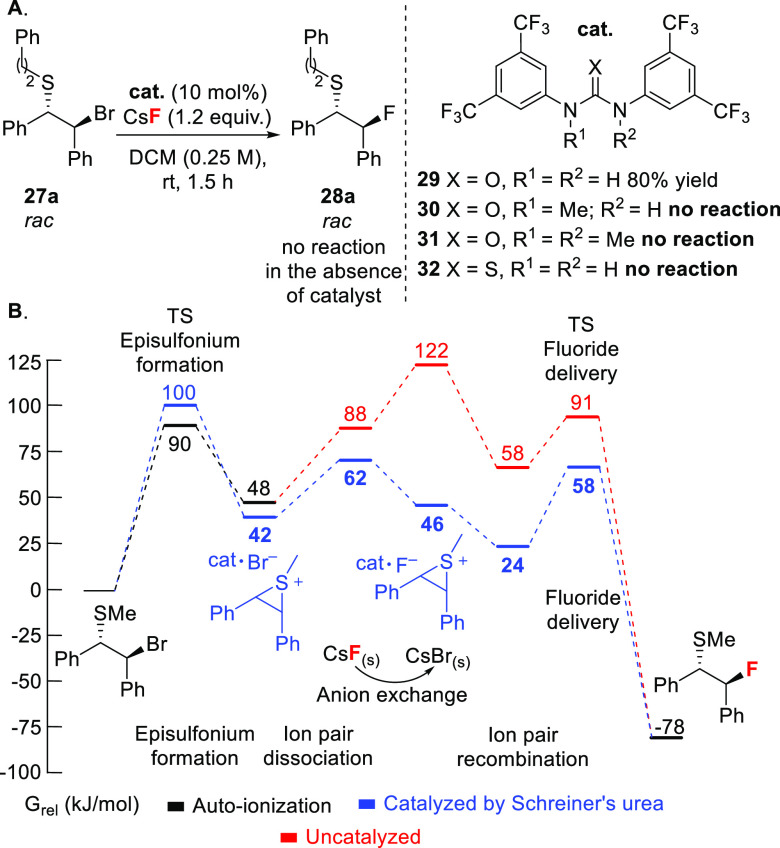
(A) HB-PTC for the Nucleophilic Fluorination of β-Bromosulfides with CsF, Catalyzed by Achiral Schreiner’s Urea, and (B) DFT-Derived Reaction Profile for a Model *S*-Methylated β-Bromosulfide

For proof of concept,^49^ we focused on the fluorination of a *meso* episulfonium which, upon ion pairing with an *in situ*-generated urea–fluoride complex, forms a complex reminiscent of the hydrogen-bonded fluoride–sulfonium pre-complex of the fluorinase enzyme.^18^ Asymmetric fluoride delivery gives access to enantiopure β-fluorosulfides of high importance in drug design.^50^ Preliminary experiments in achiral series were informative. When model stilbene-derived β-bromosulfide **27a** was reacted with CsF (1.2 equiv) in dichloromethane (Scheme 10A), no fluorination occurred, but when a catalytic amount (10 mol%) of Schreiner’s urea **29** was added,^51^ the desired alkyl fluoride **28a** was isolated in 80% yield. KF also provided the desired product but this reagent required longer reaction times. *N*-mono- and *N,N*-dimethylated H-bond donors (**30** or **31**) as well as the use of more electron-rich diarylureas diminished the yield or suppressed reactivity, indicating that hydrogen bonding is essential for fluorination to proceed. No β-fluorosulfide was obtained with thiourea **32**, a stronger H-bond donor than Schreiner’s urea **29** (p*K*_a_ ≈ 8.5 vs 13.8);^52^ in this case, alkylation of the thiourea by the episulfonium ion outcompetes fluorination because the nucleophilicity of thioureas is superior to ureas, and fluoride’s reactivity is attenuated through hydrogen bonding. Computational analysis suggests that the catalyst promotes anion exchange by preferentially stabilizing fluoride rather than bromide in solution (Scheme 10B). In the absence of catalyst, the higher lattice energy of CsF (744 kJ/mol) vs CsBr (632 kJ/mol)^16^ corroborates with an unfavorable halide exchange process (34 kJ/mol) and an overall energetic span of 122 kJ/mol. When the catalyst is present, the stronger hydrogen bonding to F^–^ over Br^–^ renders anion exchange more favorable by 16 kJ/mol. For both the catalyzed and uncatalyzed pathways, C–F bond formation is irreversible (136 and 169 kJ/mol barrier, respectively).

**Figure 1:**
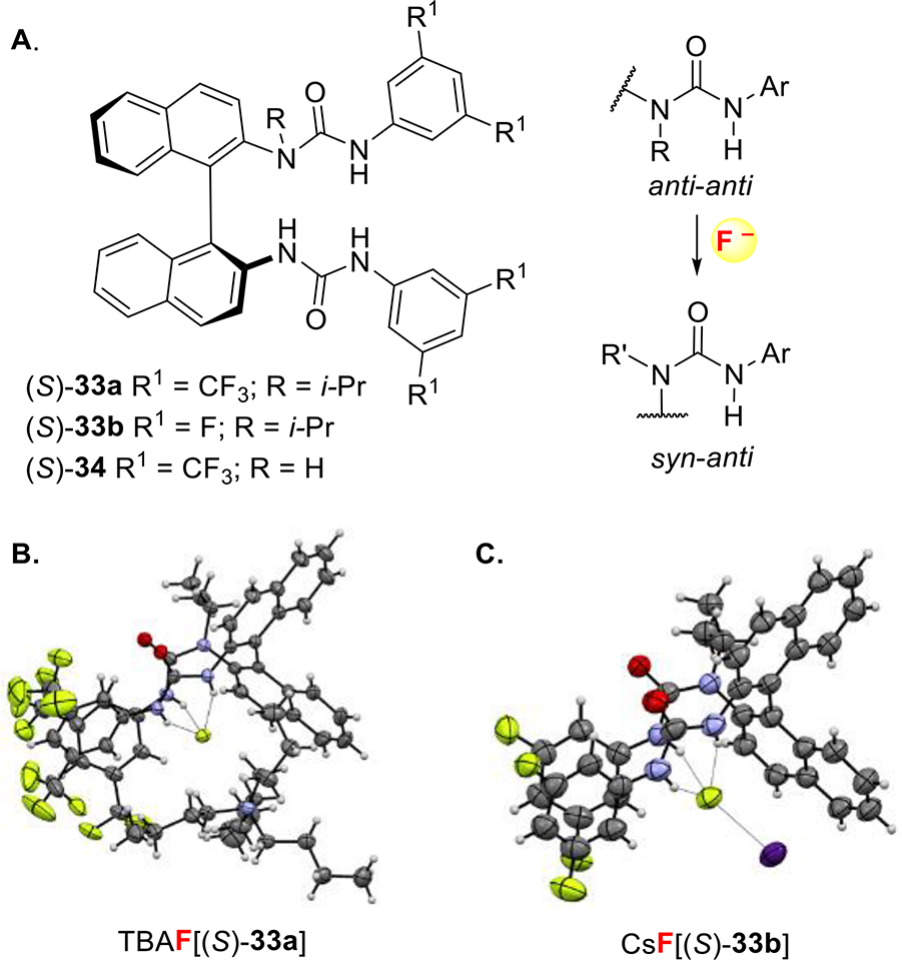
(A) BINAM-derived non-alkylated and *N*-monoalkylated bis-urea catalysts and conformational changes upon fluoride binding. (B) Single-crystal X-ray structure of TBAF[(*S*)-**33a**]. (C) Single-crystal X-ray structure of CsF[(*S*)-**33b**].

For the development of an asymmetric variant of this catalytic fluorination process, we selected BINAM-derived bis-ureas (BINAM = [1,1′-binaphthalene]-2,2′-diamine) because these chiral H-bond donors are easily prepared, and their structure modifiable through aryl substitution. *N*-Monoalkylated bis-ureas **33** (Figure 1A)^53^ were the most effective catalysts, the design of which being directly derived from computational studies. Indeed, molecular dynamics simulations in the solution phase examining the preferential binding mode of the non-alkylated BINAM catalyst **34** with fluoride indicated that not all four N–H bonds of the bis-urea catalyst need to be involved in fluoride binding. *Anti*-*anti* to *syn*-*anti* urea isomerization of the C(O)–N bond proximal to the binaphthyl core was observed, leading to a tricoordinated hydrogen-bonded fluoride complex reminiscent of the fluorinase enzyme (Figure 1A).^18^ DFT calculations confirmed that *N*-alkyl substitution reinforced the energetic preference (by 23.8 kJ/mol) for the tridentate binding mode. This was confirmed experimentally in the solid state with the X-ray structures of TBAF[(*S*)-**33a**] and CsF[(*S*)-**33b**] (Figure 1B,C) and in solution by high-resolution ^1^H NOESY.^7d^ Titrations using UV spectroscopy enabled binding constant measurements in DCMdichloromethane confirming the stronger binding of these *N*-monoalkylated catalysts to fluoride compared to bromide (*K*_a(1:1) TBAF[(*S*)-**33a**]_ = (1.7 ± 0.2) × 10^6^ M^–1^ vs *K*_a(1:1) TBABr[(*S*)-**33a**]_ = (3.3 ± 0.3) × 10^5^ M^–1^).

**Scheme 11:**
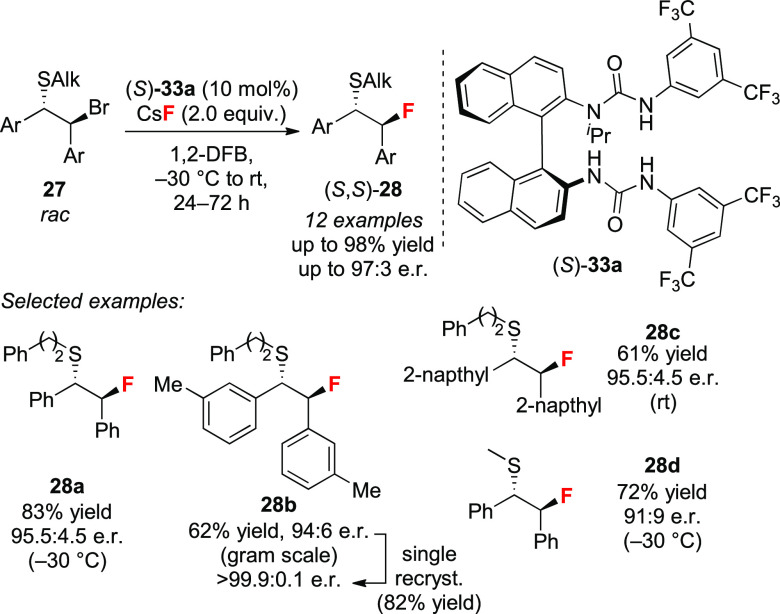
Hydrogen Bonding Phase-Transfer Catalysis for the Enantioselective Desymmetrization of Episulfonium Ions Generated from β-Bromosulfides

Experimentally, catalysts alkylated at the nitrogen proximal to the binaphthyl moiety,^54^ afforded the desired alkyl fluorides **28** in comparable yields but higher enantioselectivities. Standard reaction optimization demonstrated that halogenated aliphatic (e.g., dichloromethane) and aromatic solvents (e.g., 1,2-difluorobenzene) are best suited to enhance both reactivity and enantioselectivity. With this protocol in hand, a series of stilbene-derived episulfonium ions were desymmetrized in up to 98% yield and 97:3 e.r. using *N*-isopropylated catalyst (*S*)-**33a** (Scheme 11). A gram-scale reaction led to β-fluorosulfide **28b** as a single enantiomer (>99.9:0.1 e.r.) after one crystallization. The reaction did not require inert atmosphere or dry conditions.

### Asymmetric Nucleophilic Fluorination with KF—Desymmetrization of Aziridinium ions

**Scheme 12:**
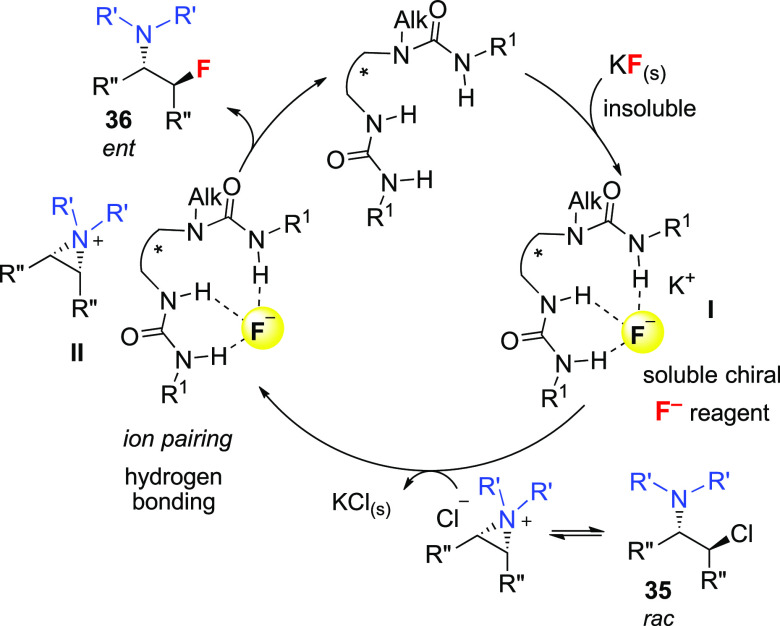
Hypothesized Catalytic Cycle for the Enantioselective Fluorination of *meso*-Aziridinium Ions Generated from β-Chloroamines Using KF under HB-PTC

Potassium fluoride is a cheap fluoride source (∼8$/mol), but its use for enantioselective fluorination under HB-PTC is challenging because its lattice energy (829 kJ/mol) is superior to that of CsF (759 kJ/mol). A study focused on KF led us to investigate the asymmetric fluorination of β-chloroamines (**35**) as *meso*-aziridinium ion precursors as a route to enantiopure β-fluoroamines (**36**),^7b^ which are of high value in medicinal chemistry (Scheme 12).^55^ In analogy with the ring-opening of episulfonium ions with CsF, we hypothesized that a chiral bis-urea hydrogen-bond donor would bring KF in solution, forming intermediate **I**, followed by the generation of the chiral ion pair **II** ({urea·F^–^}{aziridinium^+^}), with concomitant release of KCl. Irreversible formation of the C–F bond with regeneration of the catalyst would close the cycle.

**Scheme 13:**
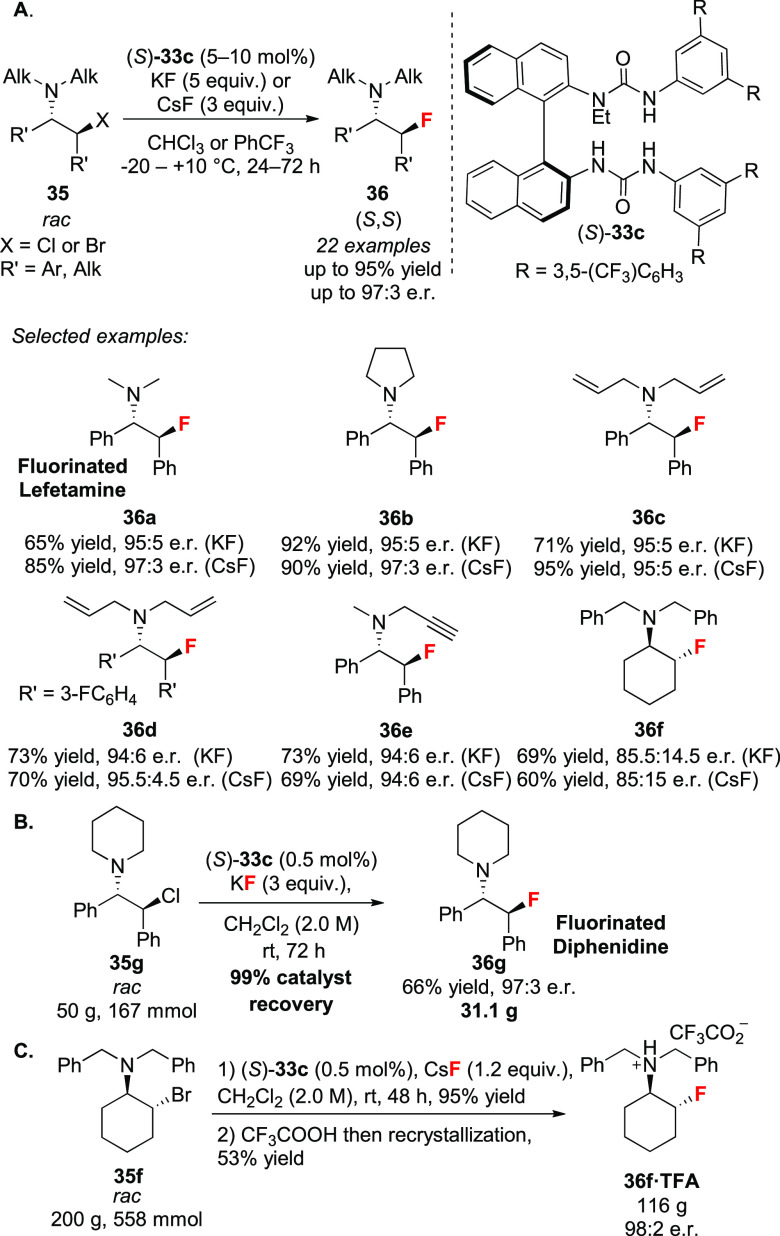
Desymmetrization of Aziridinium Ions with KF and CsF via HB-PTC: (A) Selected Examples, (B) Decagram Scale Reaction, and (C) 200 g Scale Reaction Ar = aryl; Alk = alkyl; TFA = trifluoroacetic acid.

*N*-Monoalkylated BINAM-derived bis-ureas **33** were optimal for this transformation, with the best catalyst featuring an extended polyfluorinated terphenyl moiety and an ethyl group on nitrogen ((*S*)-**33c**) (Scheme 13A). β-Chloroamines **35** with various *N*-substituents were tolerated, including *N*-allyl protection, which was cleaved under Pd(0) catalysis to afford unprotected enantioenriched β-fluoroamines. Computational DFT studies indicated that the *N*-substituents of the aziridinium ion point away from the chiral pocket of the catalyst, offering a rational for why they are well tolerated in this process. The scalability and operational simplicity of the protocol was demonstrated with a four-step reaction sequence from *cis*-stilbene to access a fluorinated analogue of the anesthetic diphenidine (**36g**)^56^ in which the last step involved a 50 g scale nucleophilic fluorination reaction of β-chloroamine (**35g**) with KF under HB-PTC in DCMdichloromethane (2.0 M) (Scheme 13B). The catalyst loading was reduced to 0.5 mol%, and the H-bond donor fully recovered after the reaction (>99%). Furthermore, the protocol did not require dry conditions or pre-treatment of KF. We also developed a protocol to synthesize decagram quantities (>30 g) of bis-urea (*S*)-**33b** and (*S*)-**33c**,^54^ and subsequently employed the latter in the 200 g scale fluorination of **35f** (this time with CsF) in a mechanical stirred 1 L glass reactor (Scheme 13C).^57^ This substrate was selected because the corresponding deprotected β-fluoroamine is a valuable building block in drug discovery.^58^ The catalyst loading was reduced to 0.5 mol% and the desired amine (*R*,*R*)-**36f** obtained in 95% yield and 81.5:18.5 e.r. Acidification and a single recrystallization (53% yield) afforded 115.7 g of (*R*,*R*)-**36f**·TFA in 98:2 e.r. Following the reaction, the catalyst was recovered and used a second time with no loss of activity or enantiocontrol. Only partial recovery of the catalyst was possible because alkylation of the catalyst with the aziridinium electrophile was observed at high concentration (2 M) in the presence of CsF. Under otherwise identical conditions, this catalyst inhibition pathway was not observed when KF was employed as fluorine source. The granulometry of CsF is a key parameter for this larger scale reaction with finely powder material (<300 μm) being required. Vacuum-dried CsF performed worse than CsF as provided from the supplier. This result underlines the tolerance of HB-PTC to trace amounts of water. Notably, deliberate addition of 10 mol% of water reduced the yields (65% vs 87%), and in the presence of 50 mol% of water, only trace of product was observed.

### Asymmetric Catalysis with Ionic Reactants—Desymmetrization of Azetidinium ions

**Scheme 14:**
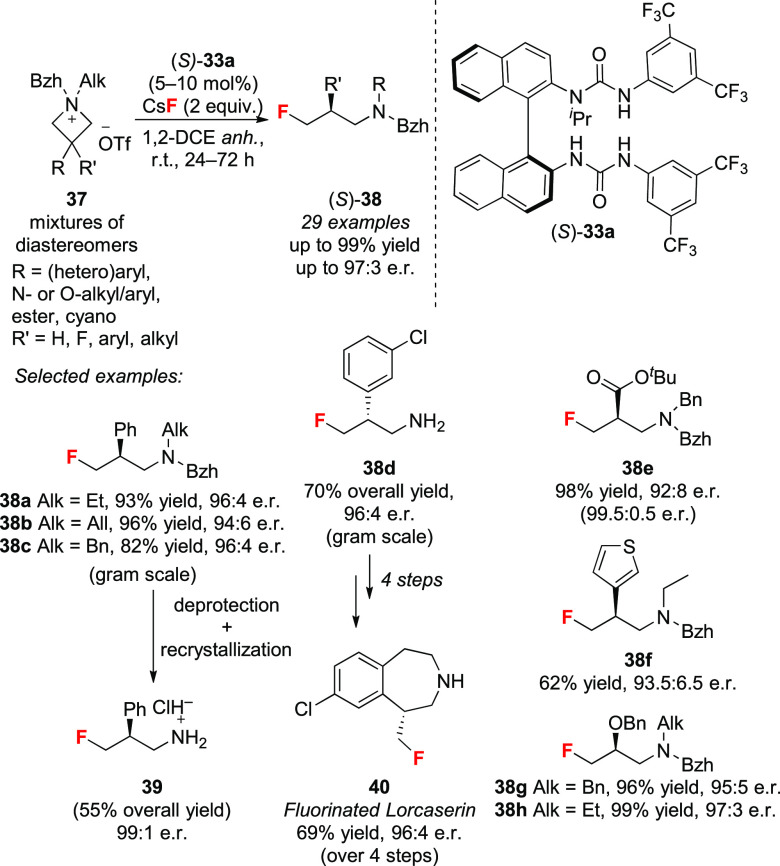
Desymmetrization of Azetidinium Salts with CsF via HB-PTC Bzh = benzhydryl; Alk = alkyl; *anh* = anhydrous

Having established the usefulness of HB-PTC for enantioselective fluorination of uncharged starting materials with CsF or KF, we studied an unexplored scenario whereby both the fluoride source and the substrate are ionic.^59^ We were drawn by this challenge as the successful desymmetrization of achiral azetidinium salts **37** would afford γ-fluoroamines (**38**), which are of relevance in medicinal chemistry (Scheme 14).^60^ This strategy presented numerous challenges, not least the fact that the azetidinium salt itself can act as phase-transfer agent for CsF or KF, possibly outcompeting the bis-urea catalyst and therefore leading to racemic products. Ammonium salts have indeed been abundantly used as phase-transfer catalysts for non-asymmetric fluorination with CsF or KF.^61^ Preliminary computational and binding studies boded well for the application of HB-PTC with ionic reactants. It was found that a neutral BINAM-derived *N*-methylated bis-urea catalyst binds CsF more strongly than a model 1,1-dimethylazetidinium ion in 1,2-dichloroethane (Δ*G*_urea_ = −69 kJ/mol vs Δ*G*_azetidinium_ = −14 kJ/mol). Furthermore, ^1^H NMR titration experiments indicated that *N*-isopropylated bis-urea (*S*)-**33a** binds fluoride more strongly than a range of possible azetidinium counter-anions: *K*_a (1:1)_: F^–^ (10^6^ M^–1^) > Br^–^ (10^5^ M^–1^) ≫ OTf^–^ (10^2^ M^–1^) > BF_4_^–^ (10^2^ M^–1^) > OTf^–^ (10^1^ M^–1^).^7a,7c^ Experimentally, diastereomeric mixtures of 3-substituted and 3,3-disubstituted azetidinium triflates (**37**) underwent asymmetric fluorination in the presence of 2 equiv of CsF and 5–10 mol% of *N*-isopropylated bis-urea catalyst (*S*)-**33a** (Scheme 14). The best results were obtained with *N*-benzhydryl azetidinium salts as starting material. Both (hetero)aryl, *O*- and *N*-substituents as well as tetrasubstituted substrates provided γ-fluoroamines in high yields and enantioselectivities. Scale up to the gram scale was successful with full recovery (>99%) of the catalyst after the reaction. The methodology was also applied to the preparation of a fluorinated analogue of FDA-approved Lorcaserin (**40**) (Scheme 14). Dry solvents increased the yields with no detrimental impact on enantioselectivity. A study aiming at comparing the reactivity of azetidinium triflates under homogeneous (2 equiv of TBAF(H_2_O)_3_ in 1,2-DCE, 24 h) and heterogeneous conditions (2 equiv of CsF, 10 mol% of (*S*)-**33c** in 1,2-DCE, 24 h) showed that the latter conditions consistently gave higher yields (20–51% vs 39–95% yield). Computational studies underlined the key role of the benzhydryl protecting group on nitrogen, which lowered the barrier to fluorination by ∼6 kJ/mol compared to a benzylated substrate; this was explained by the strain imposed by this substituent on the starting material. Furthermore, DFT-computed transition states showed that the nitrogen substituents point outside of the chiral pocket of the catalyst, underlining the unimportance of the configuration at nitrogen and supporting the enantioconvergent nature of the process.

### Mechanistic Insight on HB-PTC—Impact of Multiple Hydrogen Bonds on Fluoride Reactivity

**Figure 2:**
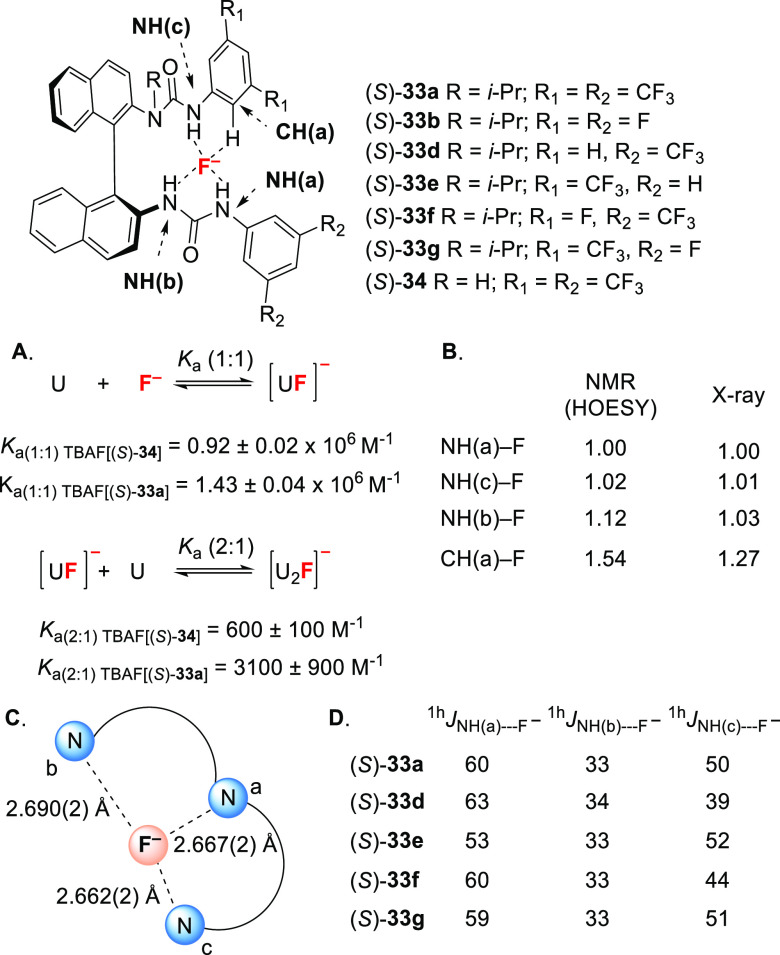
Mechanistic insight on HB-PTC. (A) Association constants for the formation of 1:1 and 2:1 TBAF(urea)_*n*_ complexes. (B) Relative H---F^–^ distances of TBAF[(*S*)-**33a**] complexes as observed by HOESY and XRD analysis. (C) N---F^–^ absolute distances from single-crystal XRD studies of TBAF[(*S*)-**33a**]. (D) Coupling constants (Hz) for selected TBAF[(*S*)-**33**] complexes.

Following the disclosure of HB-PTC, we became interested in gaining further insight on the hydrogen bonding network surrounding fluoride anions in solution, in the presence of *N*-alkylated BINAM-derived catalysts.^7d^ Such study could provide valuable information on the contribution of individual H-bond contacts on catalyst efficiency. Preliminary ^1^H NMR experiments suggested that unbound alkylated catalyst (*S*)-**33a** exists in solution as a mixture of equilibrating structures in which NH(b) is engaged in hydrogen bonding with the C=O group of the *N*-alkylated urea, an interaction which is not observed with non-alkylated (*S*)-**34**. Moreover, ^1^H NMR titration experiments of (*S*)-**34** and (*S*)-**33a** with TBAF indicate the presence of a 1:1 monomeric species which is dominant when more than one equivalent of TBAF is used. An additional dimeric or higher coordinated species (U_2_F^–^) was observed at low concentrations of fluoride. The association constants (*K*_a 1:1_ and *K*_a 2:1_) for the two species were of the order of 10^6^ and 10^2^–10^3^ M^–1^, respectively, and did not differ substantially between *N*-alkylated (*S*)-**33a** and non-alkylated (*S*)-**34** (Figure 2A). Further ^1^H NOESY experiments indicate the *N*-alkylated urea motif of bis-urea (*S*)-**33a** underwent *anti*-*anti* to *syn*-*anti* isomerization in DCMdichloromethane-*d*_2_ as predicted computationally (Figure 1A). Titration of (*S*)-**33a** with TBAF in DCMdichloromethane-*d*_2_ afforded a stable tricoordinated fluoride complex similar to the complex formed in the solid state (Figure 1B), and reminiscent of the fluoride tricoordination mode of the fluorinase enzyme.^18^ This *anti*-*anti* to *syn*-*anti* isomerization is not seen for catalyst (*S*)-**34**, for which only the two NH(s) located farther away from the binaphthyl core are involved in fluoride binding. This stark contrast between the two classes of catalysts underlines the key role of *N*-alkylation as a means to organize the coordination sphere of fluoride. Clean in-phase HSQC (CLIP-HSQC) experiments allowed the direct observation of the four nuclei involved in fluoride bonding (three NHs and F), the measurement of the corresponding coupling constants, and a measure of the length and strength of individual H-bond contacts (Figure 2B). ^1^H–^19^F HOESY experiments on TBAF[(*S*)-**33a**] showed comparable distances for NH(a)---F^–^ and NH(c)---F^–^, whereas NH(b)---F^–^ was longer (12%) and in agreement with the solid-state structure obtained by single-crystal XRD (Figure 2C). The effect of the countercation was also probed by comparing the NH---F^–^ distances of CsF[(*S*)-**33b**] in the solid state (single-crystal XRD analysis) with TBAF[(*S*)-**33b**] in the liquid state (NMR analysis, DCMdichloromethane-*d*_2_). The presence of cation−π interactions between Cs^+^ and the binaphthyl core influence the structure of the 1:1 complex by reducing the length of NH(a)---F^–^ and NH(c)---F^–^ by 3% and 10% respectively. These data suggest that the countercation indirectly influences the H-bonding network and the positioning of the fluoride in the complex. This study also showed that coupling constants serve as a useful measure of the intensity of hydrogen bonding interactions in a selected set of urea-fluoride complexes (Figure 2D). NH(a)---F^–^ was found to be the strongest interaction (highest coupling constants ^1H^*J*_NH(a)---F^–^_ = 52–60 Hz) while NH(b)---F^–^ was the weakest (^1H^*J*_NH(b)---F^–^_ = 33–34 Hz). Precise tuning of the strength of an individual H-bond contact to fluoride was possible by modifying the electronic environment of the NH bond. For example, catalysts (*S*)-**33d** and (*S*)-**33f** with more electron-rich NH(c)-aryl groups than (*S*)-**33a** were expected to have weaker NH(c)---F^–^ interaction; experimentally, this was observed with decreased ^1H^*J*_NH(c)---F^–^_ coupling constants of 39 and 44 Hz, respectively (vs 50 Hz for (*S*)-**33a**). Similarly, catalyst (*S*)-**33e** was designed to weaken NH(a)---F^–^, thereby enabling the study of the impact of this particular H-bonding interaction on the catalyst’s efficacy.

**Scheme 15:**
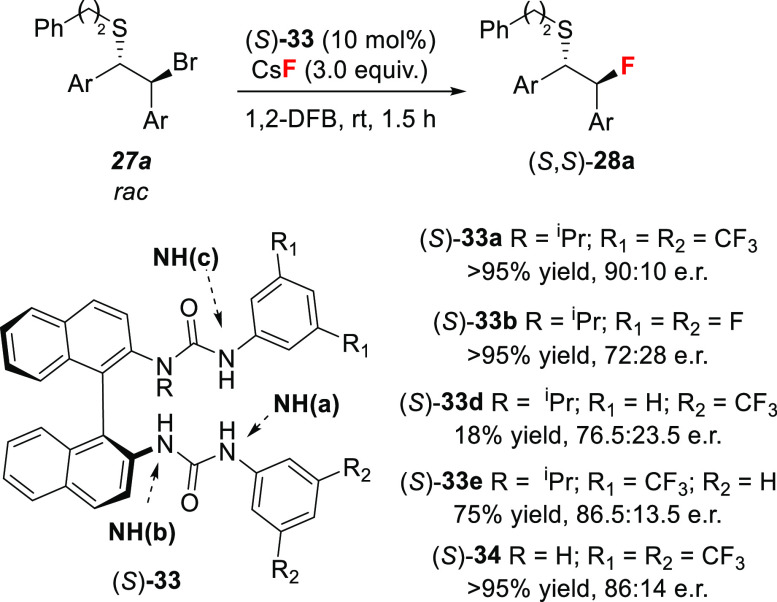
Catalytic Performance of Selected Bis-urea Catalysts in the Desymmetrization of Episulfonium Ions

With this NMR tool in hand,^7d^ we initiated a more in-depth investigation. First, we carried out the desymmetrization of episulfonium ions with stoichiometric amounts of CsF[(*S*)-**33a**] as the fluorinating reagent. This experiment afforded β-fluorosulfide **28a** in enantiomeric ratio (86.5:13.5 vs 88:12) comparable to the catalytic process (10 mol% [(*S*)-**33a**] + 3 equiv of CsF). This result supports a mechanism in which the urea–fluoride complex is responsible for fluoride delivery. A similar experiment with stoichiometric amounts of preformed TBAF[(*S*)-**33a**] and azetidinium triflate **37a** as substrate (Scheme 14) supported the same conclusions.^7c^ The fluorination of a representative β-bromosulfide with a set of bis-urea catalysts (**33**) unveiled the importance of hydrogen bonding interaction NH(c)---F^–^ for enantiocontrol (Scheme 15). Indeed, when the CF_3_ groups of (*S*)-**33a** were substituted by a single fluorine or a hydrogen, the resulting catalysts (*S*)-**33b** and (*S*)-**33d** led to a significant drop in enantioselectivity (72:28 and 76.5:23.5 vs 90:10). The weakening of hydrogen bonding interaction NH(a)---F^–^ had a much less detrimental impact on the e.r. (86.5:13.5). Furthermore, when non-alkylated catalyst (*S*)-**34** was employed, good yields (>95%) but lower enantioselectivies were observed (86:14 vs 90:10 e.r.), indicating that this catalyst was efficient in the phase transfer but the trifurcated fluoride complex is superior for enantiocontrol. Overall, these experiments suggest that each H-bond contributes to a different extent to catalyst efficiency, and therefore tuning the properties of individual H-bond contacts represents a unique yet powerful approach to design new structures with improved phase-transfer ability and enabling enhanced enantioselectivity.

## Hydrogen Bonding Phase-Transfer Catalysis with Inorganic Salts Other than Alkali Metal Fluorides

Following these studies, we sought to expand the synthetic potential of HB-PTC beyond fluorination and focused on the activation of alkali metal salts that had found limited applicability in asymmetric synthesis because of their insolubility in organic solvents.^62^ In this context, studies on KCN have been reported by Jacobsen and Song using uncharged phase-transfer organocatalysts; specific examples use KCN for the *in situ* generation of HCN in Strecker reactions using thioureas^63^ or chiral crown ethers.^5d^ Instead, we opted to demonstrate the value of HB-PTC beyond fluorination with the inorganic salt NaN_3_.

### HB-PTC with Non-fluorinated Alkali Metal Salts—Enantioselective Azidation with NaN_3_

**Scheme 16:**
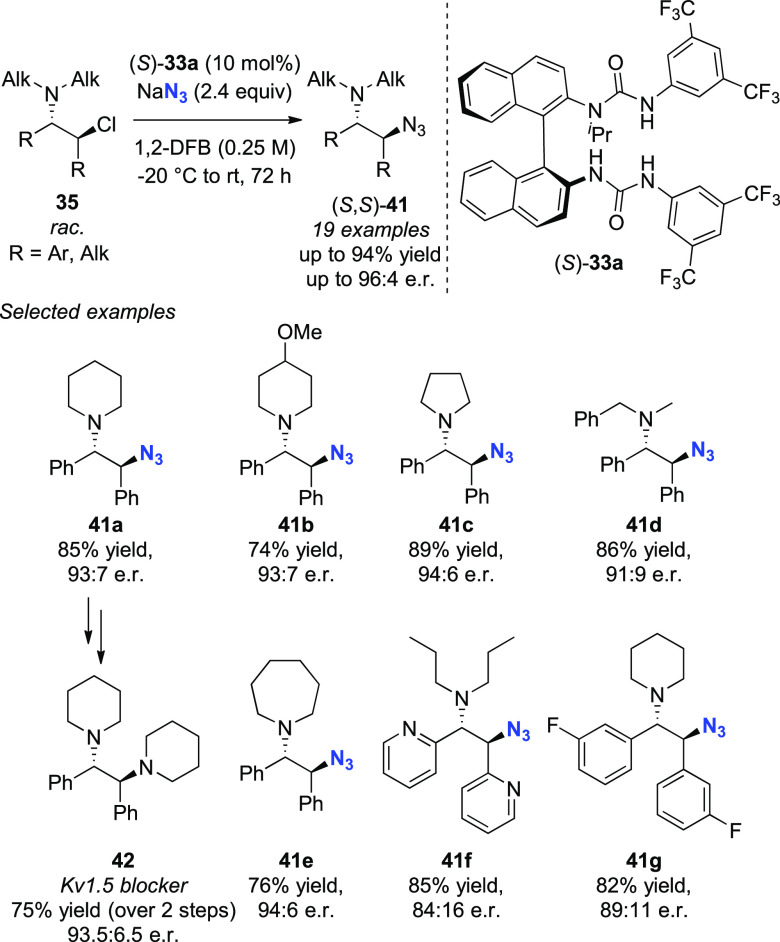
Desymmetrization of Aziridinium Ions with NaN_3_ via HB-PTC Ar = aryl; Alk = alkyl; DFB = 1,2-difluorobenzene.

Enantioenriched nitrogen-containing compounds are of particular importance in the pharmaceutical industry and are versatile chiral building blocks.^64^ Asymmetric catalytic azidations are suitable to incorporate nitrogen in organic molecules, but methodologies that rely on low-cost NaN_3_ are scarce.^65^ In our judgment, asymmetric azidation with NaN_3_ represented a valuable case study to illustrate the potential of HB-PTC because the linear azide anion represents a significant departure from spherical fluoride. Theoretical studies had suggested the use of H-bonded azides to influence regioselectivity,^66^ yet harnessing those hydrogen bonding interactions experimentally for the solubilization of NaN_3_ to enable catalytic enantioselective azidations had not been reported. Gratifyingly, we successfully subjected β-chloroamines to enantioselective azidation using NaN_3_ and 10 mol% of catalyst (*S*)-**33a** under mild conditions (−20 °C to rt) and up to the gram scale (Scheme 16).^67^ By employing a three-step azidation–reduction–alkylation sequence, 1.09 g of Kv1.5 blocker **42** was obtained.^68^ Kinetic and computational studies suggest that the rate-limiting event results in the generation of ion pair {aziridinium^+^}{(*S*)-(**33a**)·N_3_^–^}, with the progressive accumulation of NaCl being responsible for catalyst inhibition through preferential hydrogen bonding to Cl^–^.

**Figure 3:**
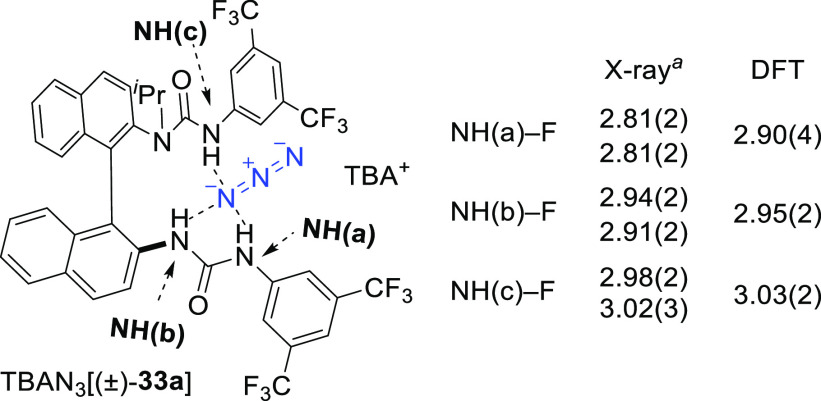
Structure and donor–acceptor (N---N) absolute distances of the TBAN_3_[(*S*)-**33a**] complex (single-crystal XRD vs lowest-energy conformer computed by DFT). ^*a*^The asymmetric unit cell contains both (*S*)- and (*R*)-enantiomers, and the measured distance is therefore provided for each enantiomer.

To shed light on the interaction between the azide anion and the H-bond donor, a series of urea–azide complexes were prepared and characterized in the solid state (single-crystal XRD analysis), and in solution and studied computationally. DFT studies carried out on the azido complex derived from bis-urea catalyst (*S*)-**33a** predicted that the most stable conformer in dichloromethane shows end-on tripodal azide binding to the three NHs of the bis-urea (Figure 3) with an arrangement similar to that with fluoride (Figure 2).^7a^ Furthermore, polarization effects induced by hydrogen bonding locate the largest residual negative charge on the coordinated nitrogen. Both ^14^N and ^15^N NMR studies in CDCl_3_ (using isotopically enriched tetrabutylammonium [1-^15^N]azide) confirmed that NH binding to azide takes place in solution. Finally, reaction of *rac*-**33a** with TBAN_3_ afforded a complex that was crystallized and characterized by XRD analysis. The coordination mode of azide to the bis-urea corroborated the lowest-energy conformer predicted computationally and confirmed the similarities with the corresponding fluoride complex (Figure 3). All three H-bond contacts to azide were longer, which suggests a weaker binding of the azide anion than fluoride. This was confirmed experimentally with ^1^H NMR titration studies of catalyst (*S*)-**33a** with TBAN_3_. The data shows that the 1:1 and 2:1 ((*S*)-**33a**)(TBAN_3_) complexes are formed with association constants of *K*_a(1:1) TBAN_3_[(*S*)-**33a**]_ = (9.14 ± 0.9) × 10^3^ M^–1^ and *K*_a(2:1) TBAN_3_[(*S*)-**33a**]_ = (1.0 ± 0.6) × 10^2^ M^–1^, respectively. This is approximately two orders of magnitude lower than those of the corresponding fluoride complexes (*K*_a(1:1) TBAF[(*S*)-**33a**]_ = (1.43 ± 0.04) × 10^6^ M^–1^ and *K*_a(2:1) TBAF[(*S*)-**33a**]_ = (3.1 ± 0.9) × 10^3^ M^–1^).^7d^

## Conclusions and Outlook

Since the first studies on the effect of hydrogen-bond donors on fluoride reactivity more than a decade ago, the field has expanded extensively, with detailed structural studies of hydrogen-bond donor fluoride complexes and the disclosure of efficient strategies to deploy alkali metal salts as fluorine sources. This research has culminated with the development of hydrogen bonding phase-transfer catalysis, which represents a novel PTC manifold which allows enantioselective fluorination using CsF or KF. The possibility of relying solely on H-bonding interactions for phase transfer opens exciting prospects in PTC. To date, this approach enabled the ring opening of *in situ*-generated aziridinium and episulfonium ions or pre-formed azetidinium salts as a route to pharmaceutically relevant fluoroamines in high enantiopurity and up to the hectogram scale. Detailed mechanistic insight suggests that precise control of the coordination sphere of fluoride through fine-tuning of the structural features of the H-bond donors may guide the design of more efficient catalysts for PTC. The challenges ahead of us are the application of HB-PTC to less activated electrophiles such as secondary or tertiary alkyl halides. Furthermore, harnessing hydrogen bonding interactions for C(sp^2^)–F bond formation with alkali metal fluorides would also open exciting prospects. To date, HB-PTC has enabled the activation of insoluble salts other than alkali metal fluorides, specifically NaN_3_. This advance creates additional opportunities and points toward the broader application of this catalytic manifold well beyond fluorinations. The asymmetric installation of other C–X as well as C–N, C–C, or C–O bonds by employing inexpensive salts as nucleophiles under PTC conditions can therefore be envisaged in the near future. In our own programme, the most pressing question is the possibility to apply HB-PTC to solubilize inorganic salts with lattice energies well above those of KF and CsF, e.g., CaF_2_. Such a challenge will likely find solutions in applying conceptual advances that go beyond PTC.
